# Parentage Analysis in Giant Grouper (*Epinephelus lanceolatus*) Using Microsatellite and SNP Markers from Genotyping-by-Sequencing Data

**DOI:** 10.3390/genes12071042

**Published:** 2021-07-05

**Authors:** Zhuoying Weng, Yang Yang, Xi Wang, Lina Wu, Sijie Hua, Hanfei Zhang, Zining Meng

**Affiliations:** 1 State Key Laboratory of Biocontrol, Guangdong Province Key Laboratory for Aquatic Economic Animals, School of Life Sciences, Sun Yat-Sen University, Guangzhou 510275, China; wengzhy5@mail2.sysu.edu.cn (Z.W.); yangy595@mail2.sysu.edu.cn (Y.Y.); wangx265@mail2.sysu.edu.cn (X.W.); wuln5@mail2.sysu.edu.cn (L.W.); huasj@mail2.sysu.edu.cn (S.H.); zhanghf27@mail2.sysu.edu.cn (H.Z.); 2 Southern Laboratory of Ocean Science and Engineering, Zhuhai 519000, China

**Keywords:** parentage assignment, relatedness analysis, single nucleotide polymorphisms, microsatellites, genotyping-by-sequencing, aquaculture

## Abstract

Pedigree information is necessary for the maintenance of diversity for wild and captive populations. Accurate pedigree is determined by molecular marker-based parentage analysis, which may be influenced by the polymorphism and number of markers, integrity of samples, relatedness of parents, or different analysis programs. Here, we described the first development of 208 single nucleotide polymorphisms (SNPs) and 11 microsatellites for giant grouper (*Epinephelus lanceolatus*) taking advantage of Genotyping-by-sequencing (GBS), and compared the power of SNPs and microsatellites for parentage and relatedness analysis, based on a mixed family composed of 4 candidate females, 4 candidate males and 289 offspring. CERVUS, PAPA and COLONY were used for mutually verification. We found that SNPs had a better potential for relatedness estimation, exclusion of non-parentage and individual identification than microsatellites, and > 98% accuracy of parentage assignment could be achieved by 100 polymorphic SNPs (MAF cut-off < 0.4) or 10 polymorphic microsatellites (mean H_o_ = 0.821, mean PIC = 0.651). This study provides a reference for the development of molecular markers for parentage analysis taking advantage of next-generation sequencing, and contributes to the molecular breeding, fishery management and population conservation.

## 1. Introduction

The development of molecular markers and sequencing technologies over the past decades has brought great innovations for aquaculture. Since mixed breeding system is often adopted in aquaculture breeding programs, accurate pedigree information is the basis of sustainable genetic selection and hatchery management [1]. Although tagging of individuals with physical tags is difficult in most fish species due to their mass spawning and tiny larvae [2,3], the introduce of molecular tools makes it feasible for fish pedigree traceability. Microsatellites, i.e., simple sequence repeats, have been classic molecular tools in parentage analysis and pedigree reconstruction in a considerably range of aquaculture fish species since its discovery in 1990s [4,5,6,7], due to their relatively small locus size, inheritance in a Mendelian fashion, codominance and high polymorphism based on the variable size of repeat units by alleles [6]. However, parentage analysis using microsatellites has suffered from genotyping errors that may cause multiple peaks, allelic dropout, null alleles or other issues, which is due in large part to the manual operation and subjective scoring process [8,9,10], and could lead to the mistakenly assignment of candidate parents. In addition, microsatellites are limited in the application in terms of genome wide trait associations or breeding value estimation, due to their relatively low density over the genome [3]. Furthermore, although Next-generation sequencing (NGS) has facilitated the mining process of molecular markers [11], genotyping of microsatellite loci can be still costly, laborious and time-consuming [12], especially when handling with a large number of individuals derived from mass spawning fish. Therefore, there is a growing tendency to seek more time- and effort-saving molecular tools among parentage related research for the few past years.

A recent rising molecular tool for parentage analysis is single nucleotide polymorphisms (SNPs), since the availability of genomic resources and multiplexed detecting methods have been greatly enhanced by NGS and bioinformatic techniques [13]. Over the last decade, SNPs have been used in parentage assignment [14], relatedness analysis [15], sibship assessment [3], population structure [16] or mating system research [17] in about 20 fish species [13]. Compared to microsatellites, SNPs are superior in their abundance over genomes [12], lower mutation rates [18], higher automation and easier standardization across laboratories [19], which effectively reduce the cost of time and genotyping errors caused by human factors. Nevertheless, the majority of SNPs are biallelic contrast to multi-allelic microsatellites, which undermines the potential of per SNP locus applying to parentage analysis because of relatively lower polymorphism and heterozygosity [20]. This problem usually can be alleviated by increasing the number of SNPs involved in analysis, and also, selecting the loci with higher minimum allele frequency (MAF) can enhance the power of markers for parentage exclusion [21]. However, the increase of loci also results in the increase of investment of time and money. Therefore, considering the trade-offs of cost and benefits when choosing the molecular tools, it is necessary to figure out the most efficient number of loci (i.e., the lowest loci number with the highest polymorphism) for conducting parentage assignment. In addition, another ongoing question is: how many SNPs are needed to match the power of one microsatellite [22]. Fernández et al. [23] suggested that 2–3 SNPs are needed to obtain an equivalent exclusion power to one microsatellite, yet Gill et al. [24] estimated that 4–5 SNPs with allele frequencies range from 0.2 to 0.8 give the same power of exclusion as for one microsatellite. Albeit the continuous attention to this issue, most of the research focused on SNP-PCR, SNP-chip or other approaches which conduct sequencing by NGS and genotyping SNPs via highly multiplexed laboratory assays [22,25,26,27,28,29], while few related reports have genotyped and filtered SNPs directly via bioinformatic pipeline based on NGS approaches such as genotyping-by-sequencing.

Genotyping-by-sequencing (GBS) is a low-coverage genotyping technique, which involves restriction enzymes to fragment genomic DNA followed by high-throughput sequencing, in order to generate high-quality polymorphism data at a relatively low per sample cost [30,31]. GBS was similar to restriction site-associated DNA sequencing (RAD-seq) but more cost-effective and less complicated [30], so that it is tailored for large-scale genotyping applications such as marker-assisted selection (MAS) and QTL mapping, if available reference genome information was provided [11]. Indeed, low-coverage GBS has been successfully used for genetic parameter estimation [32], genomic selection [33], QTL mapping [34] and genome-wide association studies (GWAS) [35] in aquaculture. It has been proposed that GBS data is one of the best options for cost-effective SNP discovery and parentage analysis [36]. Even so, the application of GBS for parentage analysis is still insufficient: only several related researches have been made for shellfish (blue mussel *Mytilus galloprovincialis*) [37], fish (Florida bass *Micropterus floridanus* [14], Australasian snapper *Chrysophrys auratus* [38], arctic charr *Salvelinus alpinus* [39]) and plant (Scots pine *Pinus sylvestris* [40], radiata pine *Pinus radiata* [41]). Despite the increase in reports of parentage analysis using GBS recently, it should still be improved for related studies to take more advantage of GBS-based techniques and bioinformatic pipelines.

Giant grouper (*Epinephelus lanceolatus* Bloch, 1790) is the largest bony fish living in coral reefs, with the largest individual recorded reaching up to 2.7 m in length and 455 kg in weight [42]. It is mainly found in tropical and subtropical waters from the Indo-Western Pacific Ocean, and because of its potential value for commerce and excellent characteristics for aquaculture, giant grouper has been a popular economic fish in the Asia Pacific region [43,44]. However, its natural populations have long been threatened by overharvest and habitat destruction, leading to a ‘vulnerable’ classification on the International Union for Conservation of Nature and Natural Resources (IUCN) Red List categories since the mid-1990s (https://www.iucnredlist.org/, accessed on 1 June 2021). Whether in conservation or aquaculture programs of this species, the application of appropriate molecular tools to trace pedigree information will help to monitor the genetic diversity of wild or cultured population and thus inform researchers of adjusting management plans. However, although 72 microsatellite markers have been developed for giant grouper so far [43,45,46,47], only a few of them have been applied in parentage related analysis [48,49]. For groupers, moreover, the development of SNP markers has only been reported for identification of growth traits in potato grouper (*E. tukula*) [50] and orange-spotted grouper (*E. coioides*) [51], while the application of SNPs for parentage analysis in groupers has not yet been reported.

In this study, we described the first GBS-based bioinformatic pipeline for discovery and screening of microsatellite and SNP loci applied to a mixed cultured family of giant grouper, in order to compare and evaluate the power of two molecular markers for testing questions about parentage and relatedness. The results testified the power of application of SNPs and microsatellites for parentage analysis in a mixed family with a certain degree of inbreeding in giant grouper. Our study contributes to providing a valuable reference for grouper conservation, fishery management and molecular breeding process.

## 2. Materials and Methods

### 2.1. Sample Preparation, GBS Library Construction and Sequencing

The giant grouper samples in this study were described in our previous work [35]. Briefly, the broodstock were derived from the South China Sea area adjacent to Hainan province, and were then transferred to the Oceans Farms Hatchery of Fisheries Research Institute of Fujian in 2014. Later in July 2017, 8 sexually mature individuals (4 females and 4 males) were collected to proceed spawning, i.e., producing F1. The newly born fish fry were cultured in 14 × 14 × 2 m^3^ (length × width × depth) concrete-walled ponds. After about 10 months of hatching, 289 individuals of F1 offspring were randomly sampled. Thumbnail-sized fin clips of both parents and offspring were collected and stored in 95% alcohol until they were placed at −80 °C, prior to DNA extraction. Genomic DNA was extracted from fin samples using TIANamp Marine Animals DNA Kit (Tiangen Biotech, Beijing, China) following the manufacturer’s protocol. The DNA extraction was qualified by 1% agarose gel electrophoresis and quantified by the NanoDrop 2000 spectrophotometers (Thermo Scientific, Waltham, MA, USA), so as to ensure the DNA concentration met the requirement of library construction (≥20 ng/μL).

The library construction and sequencing followed the original description of the authors [30,31]. In brief, 100 ng of each genome DNA extraction from all samplings (289 offspring, 8 parents) was digested with *Ecol*I and *Hae*III in 96-well plates. Barcode adaptors corresponding to each individual were designed and ligated onto the sticky ends. The DNA fragments with unique barcode adapter were pooled into libraries of 24 individuals each, purified with a PCR purification kit (NEB, Ipswich, MA, USA), and then amplified for 12 × cycles using Phusion DNA polymerase (NEB, Ipswich, MA, USA) to produce sequencing libraries. The PCR products were purified as above and quantified on a Bioanalyzer 2100 (Agilent, Santa Clara, CA, USA). The final pooled libraries were adjusted to 10 nmol, and paired-end sequencing was performed on a lane the Illumina HiSeq 2000 platform (Illumina, San Diego, CA, USA) with 150-bp reads.

### 2.2. Microsatellite Development and Genotyping

Raw sequencing data were filtered and low-quality reads were removed according to the following stringent criteria: i. reads with barcode adapter contamination; ii. reads containing ≥ 10% unidentified nucleotides (N); iii. reads with > 50% of bases with a Q value ≤ 10. After filtering, the clean data of one paternal fish was chosen for microsatellite loci identifying using MIcroSAtellite identification tool (MISA) [52], with *misa.ini* file configured as: definition (unit_size,min_repeats): 1–10 2–6 3–5 4–5 5–5 6–5; interruptions (max_difference_for_2_SSRs): 100. Modified Primer3 scripts (p3_in.pl and p3_out.pl) were applied to design batch primers, which were subsequently screened by laboratory procedures which referred to our previously published work [53] with minor modifications. In brief, 10μL volume of polymerase chain reactions (PCRs) with subject primers were carried out, and afterwards inspected by agarose gel electrophoresis and polyacrylamide gel electrophoresis (PAGE). After two rounds of screening, the selected 8 polymorphic loci alongside with 3 other loci reported in Yang’s work [46] were employed to genotyping, which was carried out in 20 μL volumes of PCR reactions (including 10 μL 2 × Taq PCR StarMix with loading dye (GeneStar, Beijing, China), 0.2 μM fluorescent-modified forward primer (FAM, HEX or ROX, synthesized by Tsingke, Guangzhou, China), 0.2 μM reverse primer, about 20 ng genome DNA and 4 μL deionized water). Thermal cycling was 94 °C for 2 min, 30 cycles of 94 °C for 30 s, 62 °C for 30 s and 72 °C for 60 s and a final extension at 72 °C for 5 min. The PCR products were analyzed on ABI3730XL genotyper with GeneScan LIZ 500 as size standard and alleles were detected by GeneMapper v3.2 software (Applied Biosystems, Thermo Scientific). All of the candidate parents and about half of the offspring were rerun to ensure the accuracy of scoring. CERVUS 3.0 [10] was used to estimate observed heterozygosity (H_o_), expected heterozygosity (H_e_), non-exclusion probability (NEP) for parentage as well as for identity across loci, and polymorphic information content (PIC). The Pearson’s correlation between locus polymorphism and non-exclusion probability has also been calculated.

### 2.3. SNP Calling and Genotyping

The filtered clean reads of each sample, as mentioned above, were aligned to our unpublished reference genome of orange-spotted grouper (*E. coioides*) using BOWTIE2 [54]. The output .sam files were transformed to binary .bam files using SAMtools [55] with *view* command, and then sorted with *sort* command. To call SNP, we indexed the reference sequences by SAMtools *faidx* command, used *mileup* and *call* command of bcftools [56] for SNP calling with consensus-caller algorithm (-c) and outputting the variants sites only (-v), and preliminarily filtered the sites with a QUAL < 20 of the raw .vcf file. We then used vcftools [57] to further filter SNPs with genotyped offspring following these stringent criteria partially referred to Zhao [14]: i. kept SNPs with coverage depth greater than 5 (—minDP 5); ii. eliminated insertion/deletion variants (—remove-indels); iii. only kept biallelic SNPs (—max-alleles 2); iv. eliminated SNPs with < 90% call rate of the population (—max-missing 0.9); v. eliminated SNPs with quality score < 98 (—minGQ 98).

Since the polymorphism of SNP loci, reflected by minimum allele frequency (MAF), can directly influence the efficiency of parentage assignment, we set a series of MAF cut-off values (0.1, 0.2, 0.3, 0.4, 0.425, 0.45 and 0.475) to figure out the lowest number with the highest polymorphism of loci for parentage assignment. SNPs deviated from Hardy-Weinberg equilibrium (HWE) with *p*-value < 0.05 (—hwe 0.05) were removed by Plink 1.9 [58]. Further, Linkage disequilibrium (LD) decay analysis using PopLDdecay [59] indicated that squared allele count correlation (*r*^2^) decreased sharply until around 10 kb, so for each SNP panel with differed MAF cut-off, we pruned one locus from each pair of loci within a 50 kb sliding window with *r*^2^ > 0.2, shifting windows by 1 bp steps, conducted by Plink 1.9. After that, we visualized the LD blocks of each locus set using Haploview 4.2 [60], which indicated that SNPs aligned to scaffolds instead of assembled chromosomes and were relatively highly linked, so we eliminated these loci in later analysis. The final obtained vcf files were converted to GENEPOP format using PGD SPIDER version 2.0.5.0 [61]. Likewise, observed heterozygosity (H_o_), expected heterozygosity (H_e_), combined non-exclusion probability across loci, and polymorphic information content (PIC) were estimated by CERVUS 3.0, and Pearson’s correlation was calculated.

### 2.4. Parentage Analysis

We conducted parentage assignment with microsatellite and SNP panels in CERVUS 3.0 and COLONY 2.0 [62]. Parentage analysis was also conducted by PAPA 2.0 [63], and since PAPA was unable to deal with the large number of SNPs, we only applied it for microsatellites. CERVUS is a classic parentage allocation analysis program based on pair-wise likelihood method, which considers the log-likelihood score of candidate parent (presented as LOD score) for specific offspring and assigns parent pair with the highest LOD score to offspring. COLONY, on the other hand, uses full-pedigree likelihood method which divides individuals into three subsamples including candidate males, candidate females and offspring, and assigns sibship and parentage simultaneously. PAPA is a parental pair allocation program based on breeding likelihood. Unlike CERVUS, it defines the likelihood of a parental pair of genotypes as the probability of it breeding the given offspring genotype among all of its possible descents, which may outperform CERVUS using data with sex-known potential parental pairs.

For microsatellites, in order to compare with SNPs and estimate optimal number alongside with polymorphism of loci, we ranked 11 microsatellite loci by PIC value, and conducted parentage assignment stepwise adding locus from the top (the most polymorphic) one to the bottom one accumulatively, which means 11 times of assignment in CERVUS. We simulated 10,000 offspring produced by 4 candidate fathers and 4 candidate mothers, with 100% parents sampled, 98% proportion of loci genotyped, 1% genotyping error rate and confidence levels assessed by LOD distribution (relaxed > 80%, strict > 95%). The minimum typed loci were set to ≥ 45% of the whole number of loci. Based on the simulation, the empirical data was tested. In PAPA, we ran loci cumulative sequence parentage allocation with a ±1 offset model (error distribution: 0.000 0.010 0.980 0.010 0.000), and correctness was assessed in simulator producing 10,000 offspring with 100 iterations. We also used COLONY to conduct parentage assignment for each microsatellite panel, assuming polygamy for both parents, using full-likelihood method with no sibship prior, updating allele frequency. The genotyping error rate and allele dropout rate was set to 0.01. Only the inferred parent pair with > 95% probability were accepted.

For SNPs, we imported each SNP panel filtered by different MAF cut-off values, as mentioned above, into CERVUS to conduct simulation and parentage assignment, with the same parameter as microsatellites except proportion of loci genotyped, which was set to the corresponding value to the specific panel. We also used COLONY to conduct parentage assignment for each SNP panel, with the same parameter described above as microsatellites.

Since true pedigree information was unknown prior to analysis, we defined the ‘standard pedigree’ as the exactly consistent pedigree assigned by 208 SNPs (derived from 0.1 MAF cut-off value) and 11 microsatellites in CERVUS (confidence > 95%) and COLONY. Individuals obtained different parentage assignment using the two types of molecular markers were excluded for the downstream analysis, because we could not ensure their true parents. We then compared each allocation result obtained from different marker panels with ‘standard pedigree’ to assess the accuracy of parentage assignment, so as to determine the most efficient loci number of SNP and microsatellite panel.

### 2.5. Relatedness Analysis

In order to compare the performance of the two types of molecular markers in distinguishing different relatedness (unrelated, half-sibling, full-sibling and parent–offspring), we also estimated pairwise relatedness between each pair of individuals using RELATED [64]. RELATED is an R package that can simulate and calculate relatedness based on seven estimators (including four non-likelihood-based and three likelihood-based ones). The expected relatedness values (*r*) are 0.5 between full-sib pairs or parent–offspring pairs, 0.25 between half-sib pairs and 0 between unrelated individuals [65]. We firstly used *compareestimators* function in this program to generate 100 pairs of individuals for estimating different relatedness, and determined that the wang [66] estimator was optimal for microsatellites and SNPs taken together. RELATED is able to generate simulated genotypes based on given population allele frequency, so for each set of SNPs and microsatellites, we generated 100 pairs of individuals for each level of relatedness using *familysim* function, and then estimated their pairwise relatedness values using *coancestry* function with the wang estimator. According to the result, we drew density plots of relatedness values for simulated pairs, so as to assess the overlap in relatedness values of individuals of different relatedness, which reflects the power of marker panels in distinguishing different pairwise relatedness. We also calculated the relatedness values between the 8 candidate parents to test their kinship.

## 3. Results

### 3.1. SNP Marker Development

After GBS sequencing, we obtained a total of approximately 13.5 million reads for 297 samples, generating about 64.4 GB of clean data. The overall alignment rate to the reference genome was 96.6%. After preliminary filtering and SNP calling with vcftools, 6,508,412 variants were kept for downstream analysis. Subsequently, we filtered SNPs following the stringent criteria described above, acquiring seven low-density SNP panels (ranged from 1013 to 5047 loci) with different MAF cut-off values. After removing the loci deviated from HWE (*p* < 0.05) and the loci in LD (*r*^2^ > 0.2), we finally retained 208, 137, 123, 100, 91, 78 and 44 loci for SNP panels with MAF cut-off values 0.1, 0.2, 0.3, 0.4, 0.425, 0.45 and 0.475, separately (Table 1).

### 3.2. Genetic Characterization and Identification Power of Microsatellite and SNP

For 289 offspring, a total of 72 alleles were detected across 11 microsatellites, with an average of 6.545 alleles per locus, ranged from 5 to 10. For SNP panels, mean H_o_ (ranged from 0.401 to 0.501) as well as mean H_e_ (ranged from 0.395 to 0.501) was lower than that of microsatellites (H_o_ ranged from 0.794 to 0.914 and H_e_ ranged from 0.672 to 0.807, separately) (Table 1). Meanwhile, mean PIC value (ranged from 0.309 to 0.375) across SNPs was also lower than microsatellites (ranged from 0.623 to 0.778) (Table 1).

**Figure 1:**
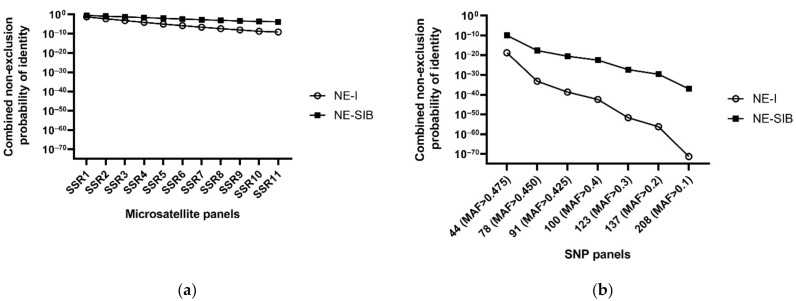
Combined non-exclusion probability for identity based on microsatellites (**a**) or SNPs (**b**). NE-I refers to the combined non-exclusion probability for identity of unrelated individual pairs, NE-SIB refers to the combined non-exclusion probability for identity of full-sibling pairs.

Identification power of both two types of molecular markers were estimated by calculating the combined non-exclusion probability for parent and individual identification, respectively. Combined non-exclusion probability for parent refers to the average probability that the given set of loci fail to exclude one or a pair of unrelated candidate parents from parentage of an arbitrary offspring. Here, the combined non-exclusion probability for one candidate parent when both parents were unknown (NE-1P) of SNPs (ranged from 2.83 × 10^−3^ to 6.53 × 10^−9^) was much lower than that of microsatellites (ranged from 0.0289 to 0.559) (Table 1). Combined non-exclusion probability for individual identification refers to the average probability that the given set of loci fail to differentiate between two randomly selected unrelated individuals or full-siblings. Likewise, the combined non-exclusion probability for individual identification of SNPs show much better identification power than microsatellites, even 44 SNPs (MAF > 0.475) can achieve several orders of magnitude higher power for individual identification (1.85 × 10^−19^ and 1.11 × 10^−10^, for unrelated and full-sibling pairs respectively) than the best value of microsatellite panels (11 loci; 8.03 × 10^−10^ and 1.57 × 10^−4^, for unrelated and full-sibling pairs, respectively) (Figure 1).

Significant negative correlation between non-exclusion probability and locus polymorphism was testified in both microsatellite and SNP markers, especially in SNPs (*p* < 0.01) (Supplementary Table S1). The polymorphic information content (PIC) and expected heterozygosity (H_e_) of loci was significantly negatively correlated with non-exclusion probability for parentage as well as for identity in both microsatellites and SNPs (*p* < 0.01), while the observed heterozygosity (H_o_) was only significantly negatively correlated with non-exclusion probability in SNPs (*p* < 0.01) (Supplementary Table S1).

### 3.3. Parentage Analysis

Due to the inconsistency or low confidence of parentage allocation of SNPs and microsatellites as well as low genotyping rate (<50%), a total of 11 individuals were eliminated (3.81%), and the remaining 278 offspring which were assigned to the same parent pairs using both 208 SNPs and 11 microsatellites (confidence > 95%), were thus retained for the downstream analysis (Supplementary Table S2). According to our result, a severe skew contribution of spawners was found, and the 278 offspring were assigned to one female half-sib family, including 173 individuals produced by dam GF01 and sire GM01, and 105 individuals produced by dam GF01 and sire GM04 respectively (Supplementary Table S2).

**Figure 2:**
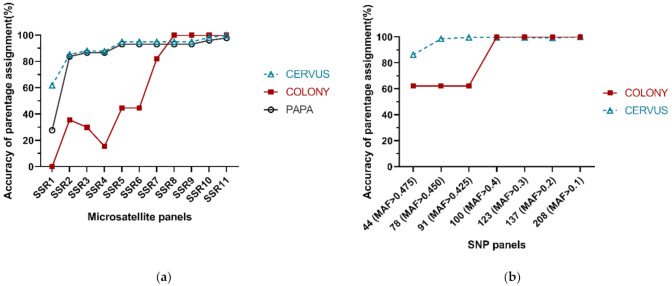
Accuracy of parentage assignment using differently sized microsatellite (**a**) and SNP (**b**) panels in different analysis programs. The accuracy was defined as the consistency derived from different marker panels compared with ‘standard pedigree’.

Overall, the accuracy of parentage assignment using both microsatellites and SNPs increases as the number of loci used increases, and ultimately reaches saturation at a certain point. For microsatellites, we analyzed differently sized panels using CERVUS, COLONY and PAPA. The accuracy of parentage assignment obtained from CERVUS was slightly better that from PAPA at the same number of loci, and both of the accuracy curves went similarly, which increases rapidly to 85.25% (83.81% in PAPA) when the second polymorphic locus gets involved, grows to 94.96% (93.17% in PAPA) at five loci, and then reaches 98.20% (96.04% in PAPA) at 10 loci (Figure 2). On the other hand, the accuracy of parentage assignment obtained from COLONY keeps lower than that from CERVUS and PAPA obviously until the eighth microsatellite loci is added in, after which it reaches a maximum of 100% (Figure 2). For SNPs, we analyzed differently sized panels using CERVUS and COLONY. The accuracy of parentage assignment obtained from CERVUS increases from 86.33% to 98.56% when MAF cut-off value was set to 0.450 (78 SNPs), while in COLONY the critical point shows at 0.4 MAF cut-off value (100 SNPs), where the accuracy increased rapidly from 62.23% to 100% (Figure 2). In general, CERVUS and PAPA performs better than COLONY when using a small number of loci, while COLONY can achieve higher accuracy when the number of loci reaches a certain amount. Further, according to our result, the accuracy of parentage assignment using around 100 polymorphic SNPs is equivalent to that of using around 10 polymorphic microsatellites.

### 3.4. Relatedness Analysis

**Figure 3:**
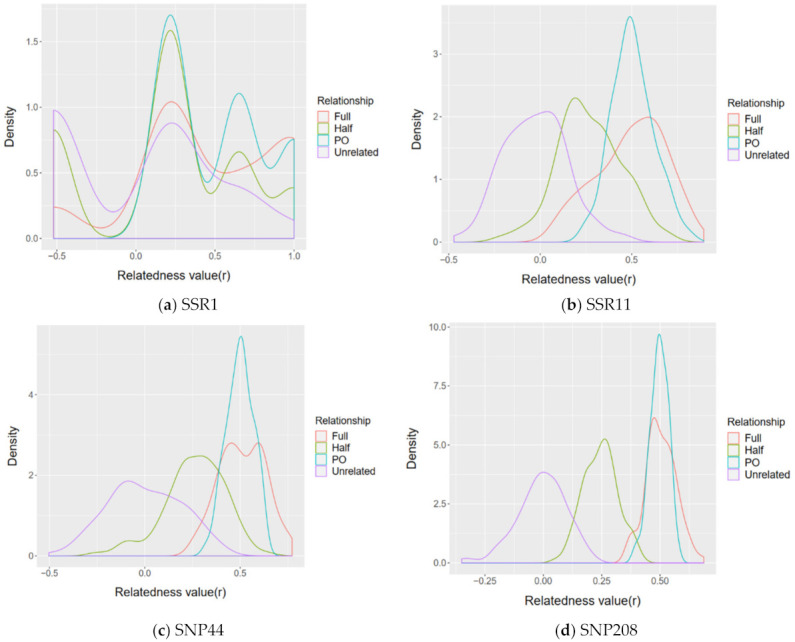
Density plots of relatedness values for simulated pairs of known relatedness (unrelated, half-sibling, full-sibling and parent–offspring) based on given population allele frequency of different marker panels: (**a**) the microsatellite panel SSR1 composed of 1 microsatellite locus; (**b**) the microsatellite panel SSR11 composed of 11 microsatellite loci; (**c**) the SNP panel SNP44 composed of 44 SNP loci (MAF cut-off > 0.475); (**d**) the SNP panel SNP208 composed of 208 SNP loci (MAF cut-off > 0.1).

The simulated estimation of relatedness value was closer to expected value when using SNP panels than that of microsatellites (Figure 3). Density plots representing histograms of the relatedness values provide informative reference for the reliability and accuracy of estimated relatedness values using specific marker panels. The overlap shows the intersection between estimated relatedness value for pairs of individuals of different relationships. Based on simulation and estimates of relatedness value, our results show that the power of differentiating relative relationships of SNP marker panels was better than that of microsatellite marker panels, even between the least number of SNPs (44) and the greatest number of microsatellites (11) (Figure 3). Regardless of the type of molecular markers, using a greater number of markers results in a less overlap between different estimated relatedness value of different relationships (Figure 3, Supplementary Figures S1 and S2).

**Figure 4:**
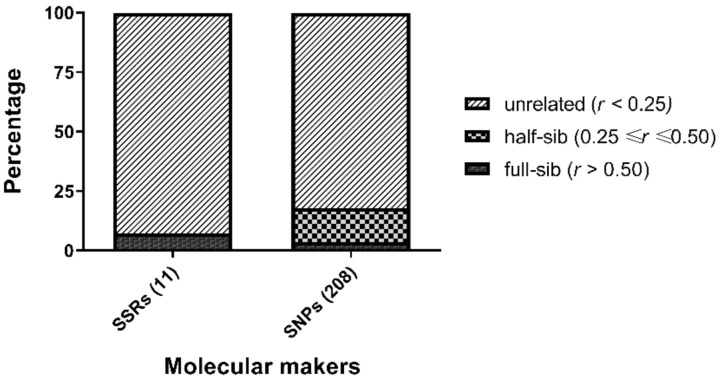
The distribution of relatedness value (*r*) of candidate parents calculated by wang estimator based on 208 SNPs or 11 microsatellites.

The relatedness values of the eight candidate parents were calculated using 11 microsatellites and 208 SNPs by wang estimator. Although most of the candidate parents seem to be unrelated (92.86% for microsatellites and 82.14% for SNPs, respectively), a certain proportion of potential kinship was found (7.14% for microsatellites and 17.86% for SNPs, respectively) (Figure 4).

## 4. Discussion

Until recent years, obtaining genetic markers was costly and laborious, and as a result, large numbers of markers were unavailable except for a few well-studied species [67]. With the rapid development of next-generation sequencing, it is easier for researchers to access to abundant genetic markers in a more cost-effective way. In terms of the advantage of GBS, Sequencing and genotyping for SNPs simultaneously offers an optional solution for relatively traditional SNP genotyping methods, which often require a mass of preparatory work at the discovery stage before genotyping of large numbers of individuals [68]. Furthermore, the relatively low coverage of GBS data enables a less computational resource-demanding pipeline for alignment with BOWTIE 2 and filtering with SAMtools and vcftools [40], and the computational time could be further reduced when conducting parentage assignment with low-density SNP panels. Although there has been a concern of the next-generation SNP genotyping methods such as missing genotype data, genotyping error and allelic dropout, several coping strategies of stringent filtering of SNPs were proposed, and a relatively stable performance of SNP panels on conducting parentage analysis were testified [12,13,14,40]. Although Andrews et al. [12] suggested that GBS was not suitable for long contig-assembly approach for designing SNP assays, Zhao et al. has illustrated an operation pipeline for low-density SNP assays designment from de novo assembly to genotyping assays utilizing Agena MassARRAY technology [14]. The bioinformatic approach we described here has testified that GBS sequencing is a cost-effective alternative for the development of microsatellites and SNPs for parentage analysis, with a low sequencing cost of lower than $40 per individual.

We assessed the polymorphism of loci to compare the potential for parentage analysis between microsatellites and SNPs. The 11 microsatellites used here represented a relatively high polymorphism and low non-exclusion probability (0.623 for mean PIC and 0.0289 for NE-1P), consistent with that of the 15 microsatellites developed by Kim et al. for giant grouper (0.511 for mean PIC and 0.02464 for NE-1P) [47]. In this study, SNPs showed a lower polymorphism yet much higher exclusion ability for both non-parentage and individual identity, suggesting a better resolution for parentage analysis compared with microsatellites. This is understandable, since SNPs are biallelic and thus lead to a lower polymorphism per locus than multi-allelic microsatellites, while the much larger number of available loci helps to accumulate their discerning ability for individuals. The significant negative correlation between non-exclusion probability and locus polymorphism in both molecular markers proved again that higher polymorphism of loci could enhance the power of markers for parentage analysis. Nevertheless, the number of loci may have a more significant effect on the power of parentage analysis than expected. Premachandra et al. [3], similarly, found the number of SNPs had a clear and major impact on the accuracy of sibship assignment, as the increase of number of SNPs led to a bigger raise in accuracy measures than that of the average MAF values. Baruch and Weller [69] conducted a series of simulations and found that non-exclusion probability decreased with increasing number of SNPs at the same level of MAF cut-off. Therefore, the advantages of SNPs in the number of available loci may have a considerable impact on their better performance of parentage analysis than microsatellites.

For captive breeding programs, it is important to maintain the genetic diversity of broodstock, and reliable relatedness parameter is needed to prevent an overrepresentation of inbreeding [15]. Moreover, appropriate demographic data for conducting classic parentage analysis is not always available for some studies, especially wild populations [68]. Thus, besides parentage analysis, the kinship of population could be illustrated by pairwise relatedness estimation. We estimated the relatedness values based on the population allele frequencies simulated from each set of SNPs and microsatellites, and found an obvious clearer resolution of distributions for individuals with different known relatedness was obtained when using SNPs or increasing the number of loci. This suggests that SNPs have the better distinguishing power of relatedness estimating than microsatellites as well. Furthermore, the assessment of genetic diversity and kinship for candidate breeders is also an important support for pre-breeding programs [70]. We also tested the relatedness values among eight candidate parent fish using 11 microsatellites and 208 SNPs, and found a certain degree of potential relatedness in at least 7.14% of parents. This result implied a potential risk of inbreeding and loss of diversity of broodstock, and also, the number of loci required for distinguishing two closer relatives would have been potentially increased [71].

To maximize the efficiency of parentage analysis, it is necessary to consider the trade-offs between number of putative primers, cost of development and panel efficacy [72]. Generally, researchers tend to resolve parentage issues by a smaller number of loci yet with higher polymorphism. We found here, for microsatellites, that a 10-loci panel (mean H_o_ = 0.821) could offer an accuracy of about 98% for parentage analysis, and meanwhile for SNPs, a panel of 100 loci (mean H_o_ = 0.500, MAF cut-off value of 0.40) was able to offer about an accuracy of 99%. This result is similar to previous reports: Kaiser et al. [36] found that 97 SNPs (mean H_o_ = 0.19) was as powerful as six multiallelic microsatellites (mean H_o_ = 0.86) for paternity assignment in black-throated blue warbler; Steele et al. [73] empirically confirmed that a panel of 95 SNPs (mean MAF = 0.34) was comparable in accuracy to a panel of 17 microsatellites (mean H_o_ = 0.74); Glaubitz et al. [20] suggested that a panel of 100 moderately polymorphic SNPs (each with a MAF of 0.20) would provide equivalent power to 16–20 independent microsatellites (each with an H_e_ of 0.75) for relationship discrimination or parentage analysis. It has also been concluded that a relatively small number of SNPs (60 to 200) can have an equivalent or better performance in parentage analysis than available microsatellites [13]. Indeed, low-density SNP panels with relatively high polymorphism are testified to be able to conduct accurate parentage analysis. Dussault and Boulding explored how different values of MAF influence the number of SNPs required for accurate parentage assignment, demonstrating that larger panels with low average MAF are needed to achieve the same accuracy as smaller panels with high average MAF [21].

According to the result of pedigree reconstruction, interestingly, we found a severe skew contribution of spawners. Among the eight communally rearing candidate parents, it seems that only two males and one female engaged in breeding, producing a large family consisting of 173 half-siblings and 105 full-siblings, respectively. The higher producing male GM01 contributed to 62.23% of the offspring, and female GF01 contributed to all of the offspring. We also found the similar skew phenomenon in previous parentage research on orange-spotted grouper (*E. coioides*) [53]. This result is unexpected, since Bright et al. [49] have described that, although a skew parental contribution exists in communally spawning giant grouper, all males and females successfully participated in production over the spawning period. The most possible explanation is that our sampling frequency of offspring was incomplete relative to the whole spawning periods, which could lead to a loss of genetic diversity and low parental contribution [49]. Moreover, since our samples come from grown-up fish instead of eggs or larvae directly, the different survival rates of progeny—which are influenced by the management practices, cannibalism and family genotype—could also result in a bias parental contribution [38,74]. More complete sampling and longer-term tracking is needed to clarify this issue.

A total of 11 individuals, which accounted for 3.81% of the total sampled offspring, were eliminated from the parentage analysis because of the inconsistency or low confidence of parentage allocation using SNPs and microsatellites. Among them, two individuals were not tested because of a low genotyping rate of 208 SNPs (< 50%), and two individuals were eliminated due to a low confidence albeit the consistent allocation between microsatellites and SNP panels. The remaining seven individuals were eliminated for inconsistence between the assignment of two molecular marker panels, while five of them showed fairly different genotype from all of the candidate parents in most loci. Besides this, we also compared the results of parentage analysis with our previous GWAS research using the same family [35]—which conducted family clustering based on identical by descent (IBD) using Plink—so as to further confirm the accuracy of parentage allocation, and found it completely consistent except for the five individuals described above. One likely explanation was that there might be individuals accidently introduced from other families during the fish culturing period. In order to figure out what happened during this process, a detailed hatchery record of spawners would be informative to provide a reference [73]. For the remaining two individuals with inconsistent allocation in two molecular marker panels, we found a divergent allocation between female GF01 and GF02 for one individual, as well as male GM03 and GM04 for another one; although considering all of the assignments including IBD clustering, the most consistent result tended to assign these two individuals to GF01 and GM04, respectively. This might be explained by the potential relatedness which were detected among candidate parents, who would share more similar genotypes and were more difficult to be distinguished, thus reducing the confidence of parentage allocation [75].

In this study, we found that the accuracy of parentage assignment using CERVUS was similar to that of PAPA, while differs from that of COLONY. Generally, CERVUS and PAPA performed better with a relatively small marker set, while COLONY offered a higher accuracy when the number of loci went to a certain amount. This result was surprising, since COLONY usually has a better performance than CERVUS in previous reports, despite a longer running time [76,77]. COLONY incorporates the sibship among putative offspring besides parent–offspring relatedness, and the likelihood of the inferred pedigree is derived from the cluster likelihoods instead of the pedigree likelihoods used in CERVUS, so that it makes more use of the pedigree information and is thus expected to perform better [78,79]. The partially decreased accuracy of parentage assignment in COLONY here might be associated with the skew group size of the largest sampled siblings, which consisted almost exclusively of two large full-sib families. As Almudevar and LaCombe deduced in their simulations, if the largest sibling group size was extremely big or the number of loci used was fairly small, the likelihood criterion might split large true clusters into smaller ones, so the clustering structure of the likelihood used in COLONY could not entirely solve the scaling problem [79]. The similar condition was described in Premachandra et al. [3], where they found the SNP pedigrees tended to split part of the full-sib families determined by microsatellites into smaller groups or produced single individuals unrelated to any other members. Moreover, the default low genotyping error rate of 0.01% to 1% seemed too low even for a relatively low-density SNP panel [80], allowing only for ≤ 2 genotyping errors for a whole panel of 208 SNPs, which might cause interference to a certain extent and lead to the erroneous assignment of parents. Hall et al. [40] detected a genotyping error rate of 5% in GBS data, which suggested a finer selection of SNP loci would be needed to alleviate the consequence brought by such kind of error.

Another possible factor that might reduce the genotyping rates could come from the bioinformatic pipeline, although we have filtered SNPs following a stringent criterion including eliminating loci deviated from Hardy–Weinberg equilibrium or with high linkage (*r*^2^ > 0.2) [14]. Since the reference genome we used for alignment was from orange-spotted grouper (*E. coioides*), and considering the DNA sequence divergence between orange-spotted grouper and giant grouper, it might be more ideal to refer to a genome of giant grouper for this study.

## 5. Conclusions

Here, we described a GBS-based bioinformatic pipeline for the development of microsatellite as well as low-density SNP marker panels for giant grouper for the first time, and testified their performance on parentage and relatedness analysis. The power of SNPs for exclusion and relatedness estimation was better than that of microsatellites, and > 98% accuracy of parentage assignment could be achieved by around 100 polymorphic SNPs or 10 polymorphic microsatellites. Taking advantage of the next-generation sequencing like GBS, pedigree reconstruction could be conducted with a considerably low cost of money and time.

## Figures and Tables

**Table 1:** Genetic characterization and combined non-exclusion probability for parent of microsatellites and SNPs.

Molecular Marker	*n*	MAF Cut-Off	Proportion of Loci Typed	H_o_	H_e_	PIC	NE-1P	NE-PP
Microsatellites	11	-	1	0.794	0.672	0.623	0.0289	2.13 × 10^−5^
	10	-	1	0.821	0.698	0.651	0.0316	2.97 × 10^−5^
	9	-	1	0.820	0.709	0.661	0.0395	6.69 × 10^−5^
	8	-	1	0.834	0.720	0.673	0.0500	1.48 × 10^−4^
	7	-	1	0.852	0.734	0.687	0.0637	3.40 × 10^−4^
	6	-	1	0.871	0.743	0.697	0.0854	8.50 × 10^−4^
	5	-	1	0.904	0.752	0.708	0.118	2.25 × 10^−3^
	4	-	1	0.911	0.761	0.719	0.166	6.16 × 10^−3^
	3	-	1	0.912	0.771	0.732	0.240	0.0179
	2	-	1	0.914	0.790	0.757	0.348	0.0524
	1	-	1	0.903	0.807	0.778	0.559	0.201
SNPs	208	>0.1	0.941	0.401	0.395	0.309	6.53 × 10^−9^	1.15 × 10^−25^
	137	>0.2	0.937	0.481	0.478	0.362	5.00 × 10^−7^	9.36 × 10^−20^
	123	>0.3	0.935	0.495	0.491	0.370	1.40 × 10^−7^	3.89 × 10^−18^
	100	>0.4	0.936	0.500	0.498	0.373	1.91 × 10^−6^	5.24 × 10^−15^
	91	>0.425	0.936	0.501	0.499	0.374	5.93 × 10^−6^	9.70 × 10^−14^
	78	>0.450	0.936	0.501	0.500	0.374	3.18 × 10^−5^	6.80 × 10^−12^
	44	>0.475	0.939	0.496	0.501	0.375	2.83 × 10^−3^	4.90 × 10^−7^

Legend: *n*, the loci number; H_e_, expected heterozygosity; H_o_, observed heterozygosity; PIC, polymorphic information content; NE-1P, average non-exclusion probability for one candidate parent when both parents were unknown; NE-PP, average non-exclusion probability for a candidate parent pair when both parents were known.

## Data Availability

The data presented in this study are openly available in the DDBJ sequence read archive (DRA), DRA Accession no. DRA012199.

## Supplementary Materials

The following are available online at https://www.mdpi.com/article/10.3390/genes12071042/s1, Figure S1: Density plots of relatedness values for simulated pairs of known relatedness based on microsatellite panels (from SSR2 to SSR10), Figure S2: Density plots of relatedness values for simulated pairs of known relatedness based on SNP panels (from SNP78 to SNP137), Table S1: The Pearson’s correlation between non-exclusion probability and polymorphism of microsatellite and SNP marker, Table S2: The results of parentage assignment based on 11 microsatellites and 208 SNPs in CERVUS and COLONY.
