# Supplementary material for: Parentage Analysis in Giant Grouper (Epinephelus lanceolatus) Using Microsatellite and SNP Markers from Genotyping-by-Sequencing Data
Source: Genes (Basel). 2021 Jul 5;12(7):1042. doi: 10.3390/genes12071042 (PMC8304347; doi:10.3390/genes12071042)
Supplement: Supplementary file 1 [file genes-12-01042-s001.zip › Supplementary_Materials_Figure_S1&S2&Table_S1.pdf]

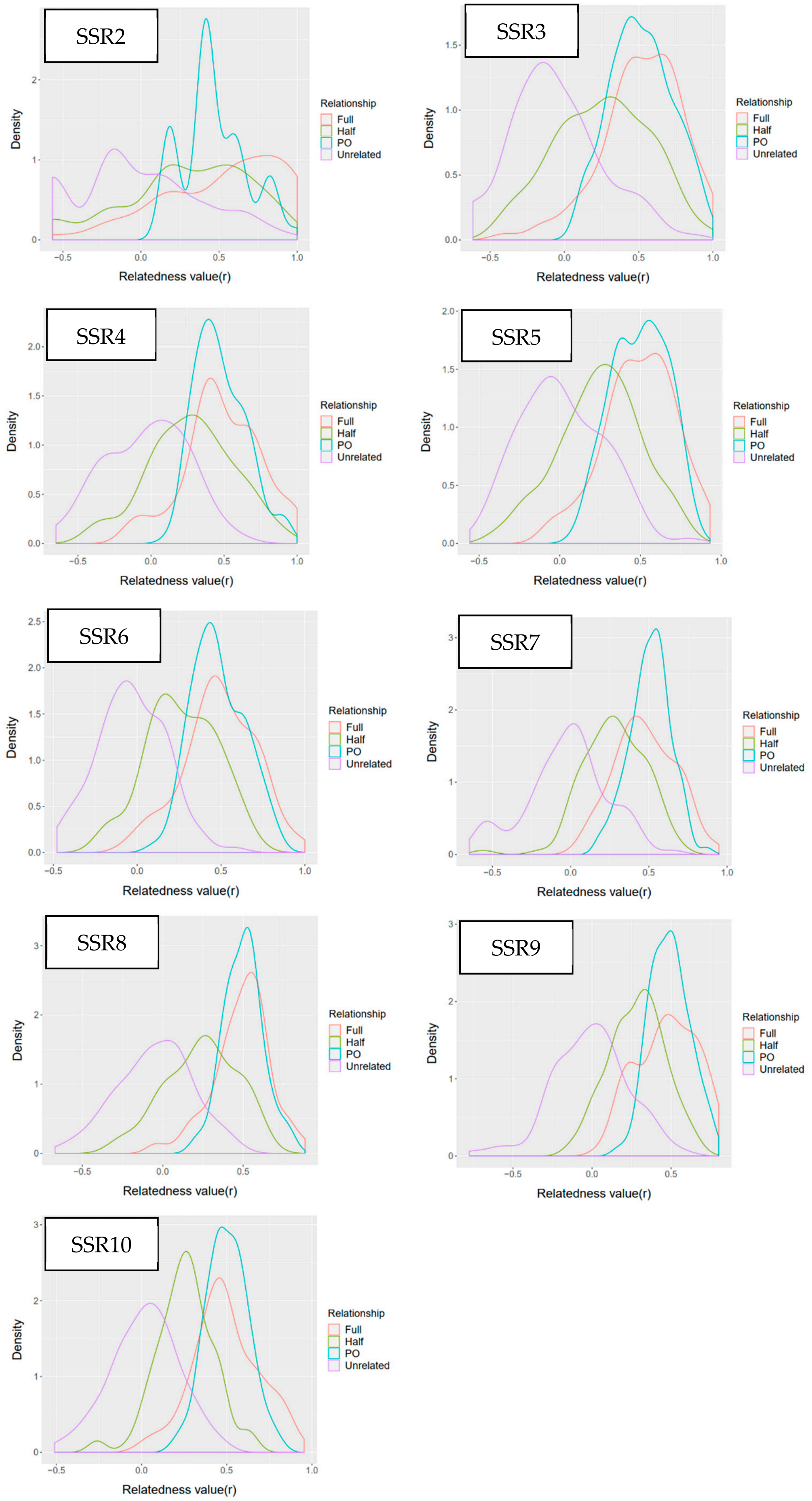

Figure S1. Density plots of relatedness values for simulated pairs of known relatedness based on microsatellite panels (from SSR2 to SSR10).

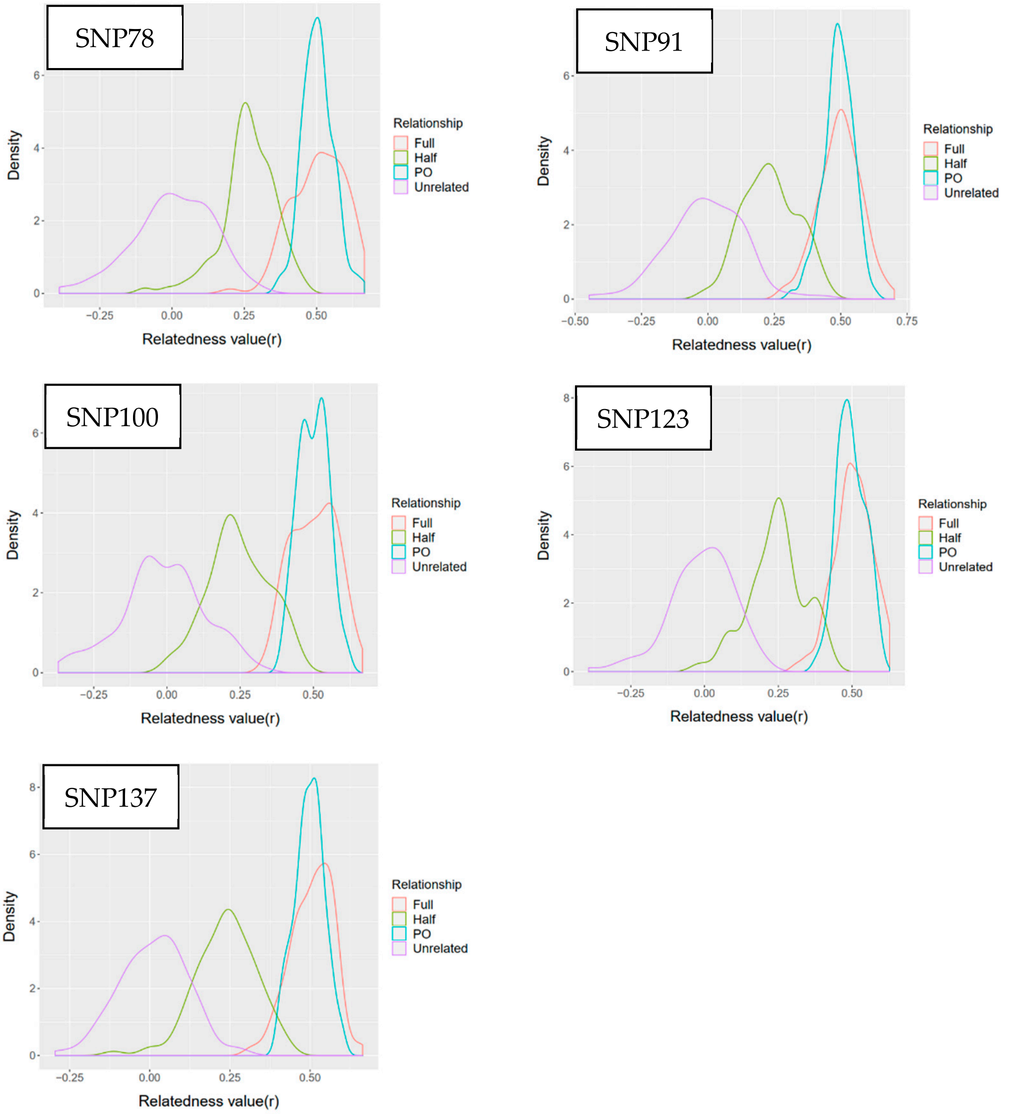

Figure S2. Density plots of relatedness values for simulated pairs of known relatedness based on SNP panels (from SNP78 to SNP137).

Table S1. The Pearson's correlation between non-exclusion probability and polymorphism of microsatellite and SNP marker.

Molecular marker	NEP	H_o	H_e	PIC
Microsatellites (11)	NE-1P	-0.009	-0.990**	-0.982**
	NE-PP	0.030	-0.974**	-0.998**
	NE-I	0.039	-0.954**	-0.994**
	NE-SIB	0.020	-0.972**	-0.999**
SNPs (208)	NE-1P	-0.972**	-0.997**	-0.992**
	NE-PP	-0.973**	-0.998**	-1.000**
	NE-I	-0.971**	-0.996**	-0.999**
	NE-SIB	-0.975**	-1.000**	-1.000**

Legend: NEP, non-exclusion probability; He, expected heterozygosity; Ho, observed heterozygosity; PIC, polymorphic information content; NE-1P, average non-exclusion probability for one candidate parent when both parents were unknown; NE-PP, average non-exclusion probability for a candidate parent pair when both parents were known; NE-I, average non-exclusion probability for identity of unrelated individual pairs; NE-SIB, average non-exclusion probability for identity of full-sibling pairs.
